# More than Attentional Tuning – Investigating the Mechanisms Underlying Practice Gains and Preparation in Task Switching

**DOI:** 10.3389/fpsyg.2017.00682

**Published:** 2017-05-10

**Authors:** Mike Wendt, Stina Klein, Tilo Strobach

**Affiliations:** Department of Psychology, Faculty of Human Sciences, Medical School HamburgHamburg, Germany

**Keywords:** task switching, preparation, switch costs, training, executive functions

## Abstract

In task switching, participants perform trials of task repetitions (i.e., the same task is executed in consecutive trials) and task switches (i.e., different tasks are executed in consecutive trials) and the longer reaction times in switch trials in comparison to these times in repetition trials are referred to as switch costs. These costs are reduced by lengthening of an interval following a cue that indicates the upcoming task; this effect demonstrated effective task preparation. To investigate the role of task switching practice for these preparation effects and task switch costs, we applied a task switching paradigm, involving two digit classification tasks, in six successive practice sessions and varied the length of the preparation interval. To further examine practice-related processing alterations on preparation, particularly concerning the focusing of visual attention and control of response competition, we added an Eriksen flanker task in the initial and the final session. Unlike the two digit tasks, which were always validly cued, the Eriksen flanker task occurred randomly after a cue that indicated one of the other two tasks (i.e., invalid task cuing). The results showed that, in the initial session, task switch costs for the digit tasks were reduced after a long preparation interval but this reduction disappeared after practice. This finding is consistent with the assumption of practice-related enhancement of preparation efficiency concerning non-perceptual task processes. Flanker interference was larger after preparation for a task repetition than for a task switch and (regarding error rates) larger in the final than in the initial session. Possible mechanisms underlying these attentional modulations evoked by task-sequence-dependent preparation and by task switching practice are discussed.

## Introduction

To investigate cognitive flexibility, researchers often apply task switching situations. In these situations, participants execute two different tasks in varying sequences, usually on the same set of target stimuli. These tasks are frequently afforded by distinctly different perceptual dimensions thereof, such as when participants switch between a shape classification and a color classification when presented with colored geometrical shapes. In the *task-cuing procedure*, the two tasks are presented in random order and participants are informed about the identity of the upcoming task by a cue that precedes or accompanies the presentation of the target stimulus (e.g., Meiran, 1996). Switching between tasks (i.e., executing a different task than on the directly preceding trial) incurs a cost in reaction times (RTs) and sometimes error rates in comparison to task repetitions (i.e., executing the same task on successive trials). These costs are referred to as *(task) switch costs* (overview in Monsell, 2003; Kiesel et al., 2010; Vandierendonck et al., 2010).

## Task Preparation

Because participants are informed about the identity of the upcoming task by the task cue, a manipulation of the length of the cue-target interval (CTI) produces different amounts of processing time for task-specific preparation. Performance usually benefits from an increase of the CTI, more so on task switch trials in comparison to repetition trials, resulting in a reduction of the switch costs (e.g., Meiran, 1996). This reduction at long in contrast to short CTIs has been referred to as the *Reduction In Switch Cost (RISC) Effect* (Liefooghe et al., 2009).

The RISC Effect in the task-cuing procedure has been accounted for in terms of more effective task preparation in task switch trials, suggesting some form of advance task-set reconfiguration not necessary in task repetition trials (Rogers and Monsell, 1995). Although various suggestions have been made regarding specific components of this reconfiguration (for an overview, see Kiesel et al., 2010), little consensus has been reached so far. However, a coarse distinction can be made concerning preparatory attentional weighting of perceptual dimensions (i.e., biasing processing toward the target stimulus dimension of the upcoming task, e.g., Meiran, 2000; see also Müller et al., 2003; Lien et al., 2010) and preparation of non-perceptual task processes, such as increasing the readiness of the application of task-specific stimulus–response transformation rules (e.g., Mayr and Kliegl, 2000). Whereas attentional weighting may facilitate performance in case the component tasks are associated with distinct perceptual target dimensions (e.g., color vs. shape classification tasks), non-perceptual preparation may also be applied in such situations. Therefore, preparation effects observed when tasks are associated with different stimulus dimensions, are ambiguous regarding a perceptual vs. non-perceptual preparation locus.

In contrast, attentional weighting cannot be applied when the component tasks are not afforded by different perceptual target dimensions. A frequently implemented example of the latter situation involves switching between purely semantic classification tasks, such as when participants judge the magnitude vs. the parity of stimulus digits (e.g., Sudevan and Taylor, 1987; Schuch and Koch, 2003; Kiesel et al., 2007). Because performance benefits when the CTI is increased (i.e., the RISC Effect occurs) in such situations (e.g., Schuch and Koch, 2003) it can be concluded that task-specific preparation is not confined to re-adjustment of attentional weights assigned to perceptual dimensions, but rather to preparation of non-perceptual task processes.

## Task Switching Practice

Several studies have demonstrated that switch costs are reduced with practice distributed over two or more experimental sessions (Rogers and Monsell, 1995; Kray and Lindenberger, 2000; Cepeda et al., 2001; Kray and Eppinger, 2006; Karbach and Kray, 2009; Zinke et al., 2012) with some studies showing an extreme reduction of such costs to (still statistically significant) 6, 8, or 20 ms (Berryhill and Hughes, 2009; Strobach et al., 2012). In some studies, the reduction of switch costs after practice occurred under conditions of comparably short preparation intervals (e.g., Rogers and Monsell, 1995; Meiran et al., 2000; Minear and Shah, 2008), indicating that the practice-related facilitation of processing in switch trials does not depend on time-consuming preparatory processes. Extending these findings, Meiran et al. (2000) observed a three-way interaction involving trial type (i.e., task repetition vs. task switch), preparation interval (i.e., CTI), and practice, reflecting a practice-induced reduction of the RISC Effect. That is, switch costs were larger in trials associated with a short than with a long preparation interval in the first session but less so in the second session (see Meiran, 1996, for a similar finding, obtained during the course of a single experimental session). In a study of Cepeda et al. (2001), the reduction of the RISC Effect after practice failed to reach statistical significance but a significant reduction of the preparation benefit in task switch trials compared to (repetition) trials from single-task blocks (i.e., blocks with only one component task) was found. A plausible explanation of these practice findings is to assume that task switching practice results in enhanced efficiency of task (switch) preparation (i.e., less time needed to achieve a prepared state after practice). Because in the study of Meiran et al. (2000) no analogous effect was observed regarding the interval between the response in one trial and the task cue in the following trial, task switching practice does not seem to result in speed-up passive decay of the previously applied task-set.

Noteworthy, in both studies, Meiran et al. (2000) and Cepeda et al. (2001), participants switched between tasks that differed regarding their perceptual target dimensions. More precisely, in Meiran et al.’s study, participants judged the vertical vs. horizontal displacement of a stimulus in a 2 X 2 grid, whereas in Cepeda et al.’s study participants were presented strings of repetitive digits (e.g., *333*, *3333*, *22*, or *2222*) and were either required to count the number of or to identify the elementary digits. Task preparation effects in these previous studies may thus be brought about by perceptual preparation. That is, task switching practice may have resulted in a speed-up of preparatory re-adjustment of attentional weights given to the upcoming task’s stimulus dimension, leaving other task-specific mental operations unaffected. Such attentional biasing may constitute a powerful means of task selection (e.g., Meiran et al., 2008). On the other hand, it does not provide an universal method of dealing with alternative task demands as it can only be applied when the tasks to-be-switched are associated with differing perceptual stimulus dimensions. In the current study, we aimed to extend previous findings to the preparation of non-perceptual task processes. To this end, we provided participants with task switching practice of six sessions for a combination of tasks (i.e., magnitude vs. parity judgements) that were not associated with perceptually different stimulus dimensions, and varied the preparation interval (i.e., CTI).

A second indication of interference between task-sets, in addition to the switch costs, is usually observed when the tasks switched between involve the same set of stimuli and motor responses. In that case, stimuli can be categorized depending on whether they afford the same motor response in both tasks or whether they afford different responses, referred to as congruent and incongruent, respectively. For illustration, consider switching between parity and magnitude judgments with a left-sided key press response to indicate that the stimulus digit is odd or smaller than 5 and a right-sided key press response to indicate that the stimulus digit is even or larger than 5. With such an arrangement the digit *3* would be congruent (i.e., left key responses in magnitude and parity judgment tasks) whereas the digit *7* would be incongruent (i.e., right key response in magnitude judgment task and left key response in parity judgment task). *Congruency effects*, that is, worse performance in trials involving incongruent compared to congruent stimuli, as observed in many studies (e.g., Rogers and Monsell, 1995; Meiran, 1996; Kiesel et al., 2007), thus reflect some kind of application of the stimulus–response translation rules of the irrelevant task to the current stimulus (Meiran and Kessler, 2008; Wendt and Kiesel, 2008). Interestingly, contrasting with task switch costs, congruency effects are often not reduced when the CTI is increased (e.g., Rogers and Monsell, 1995; Meiran, 1996), suggesting that increased preparation is not associated with enhanced shielding of task performance against this form of task interference. In the current study, we used overlapping sets of stimuli and responses which allowed us to examine the role of extended practice on task preparation and congruency effects.

In addition to assessing performance after practice in the practiced tasks we employed a probe task method to investigate possible practice-related alterations in task preparation. Specifically, we intermixed trials of a third task which was not presented in the practice sessions. This (probe) task involved a different set of stimuli and occurred with equal probability after a cue indicating the magnitude or parity judgment tasks. In such situations, processing of the probe task should suffer from malpreparation (i.e., from preparation for the task invalidly indicated by the cue, e.g., Hübner et al., 2004; Wendt et al., 2012), thus more advanced task preparation might evidence itself in modulated performance in probe task trials. More specifically, assuming that longer CTIs are used for more advanced task-set reconfiguration one would expect a disadvantage of probe task performance after longer CTIs, and assuming that task switching practice results in a speed-up of task-set reconfiguration processes during preparation, a similar disadvantage should emerge after a short CTI.

This probe task method offers the opportunity to investigate specific aspects of processing characteristics by choosing a probe task that is associated with well-established and well-understood behavioral effects. Modulations of these effects by task switching practice may reveal specific processes or representations affected by the practice experience. As a first step in this direction, we used an Eriksen flanker task (Eriksen and Eriksen, 1974) as probe task. This task is widely considered diagnostic for the occurrence of competition between response representations evoked by the target stimulus and by surrounding irrelevant stimuli, referred to as flankers (e.g., Gratton et al., 1992; overview in Eriksen, 1995). Intermixing trials of this probe task thus allowed us to assess the degree of response competition evoked by irrelevant stimuli in an unexpected task as a function of task preparation in various conditions of the task switching context (i.e., task repetition and task switch, short and long CTI, before and after practice). Because the resulting strength of response competition is thought to depend on a set of perceptual-cognitive processes, collectively referred to as selective attention, differences in the response competition effect may be informative about the attentional set in these situations. We will consider more specific suggestions regarding the underlying attentional processes in the Section “Discussion.”

In summary, the current study was designed to investigate the effect of task switching practice on non-perceptual processes of task preparation. To this end, we conceptually replicated experiments of Meiran et al. (2000) and Cepeda et al. (2001) using a combination of tasks that did not differ regarding their perceptual dimensions. Assuming that task switching practice results in a speed-up of (non-perceptual) task preparation, we expected to observe a practice-related reduction of the RISC effect. In addition, we explored practice effects of task preparation on task representations by presenting an unexpected probe task that allowed us to assess attentional aspects of stimulus–response processing (i.e., variations in the degree of response competition evoked in an unexpected flanker task). From a broader perspective, the current practice study thus investigates flexible action selection according to one’s current task goal, a hallmark of *executive functioning*, and it’s plasticity to the effects of practice (e.g., Karbach and Verhaeghen, 2014; Strobach et al., 2014).

## Materials and Methods

### Participants

Twenty students of the Medical School Hamburg (17 female) participated in the experiment in exchange for course credit. They ranged in age from 21 to 31 years. All participants had normal or corrected to normal vision by self-report.

### Apparatus and Stimuli

Stimulus presentation and RT measurement were performed with a PC. The digits *1* to *9* except *5* were used as stimuli for the magnitude and the parity task. They were displayed on a 22″ monitor with a refresh rate of 60 Hz, viewed from a distance of about 60 cm. All digits were presented in white color on a black background, in the center of the screen. The digits extended 0.6 cm (approximately 0.6°) vertically and from 0.3 to 0.4 cm horizontally (approximately 0.3°–0.4°). Colored disks with a diameter of 0.6 cm (approximately 0.6°), presented in the center of the screen, were used as task cues. A blue disk indicated the magnitude task, and a red disk indicated the parity task. On flanker task trials, three arrows, extending in the horizontal dimension, were presented. One of the arrows (i.e., the target) was presented in the center of the screen, whereas the other two arrows (i.e., the flankers) surrounded the central arrow symmetrically in the vertical dimension. (All three arrows were horizontally aligned.). The two flanker arrows of a trial always pointed into the same direction and either in the same direction as the target arrow (i.e., compatible) or in the opposite direction as the target arrow (i.e., incompatible). A target-flanker ensemble extended 1.3 cm (approximately 1.2°) vertically and 0.7 cm (approximately 0.7°) horizontally.

Responses were given by pressing the Y key (left) and the M key (right) on a standard QWERTZ-keyboard. Participants pressed the response keys with the index fingers of their left and right hand. In the magnitude task, participants pressed the left key to indicate smaller than 5 and the right key to indicate larger than 5. In the parity task, participants pressed the left key to indicate even and the right key to indicate odd. In the flanker task, participants pressed the left key and right key to indicate that the target arrow pointed to the left and the right, respectively.

### Procedure

**FIGURE 1:**
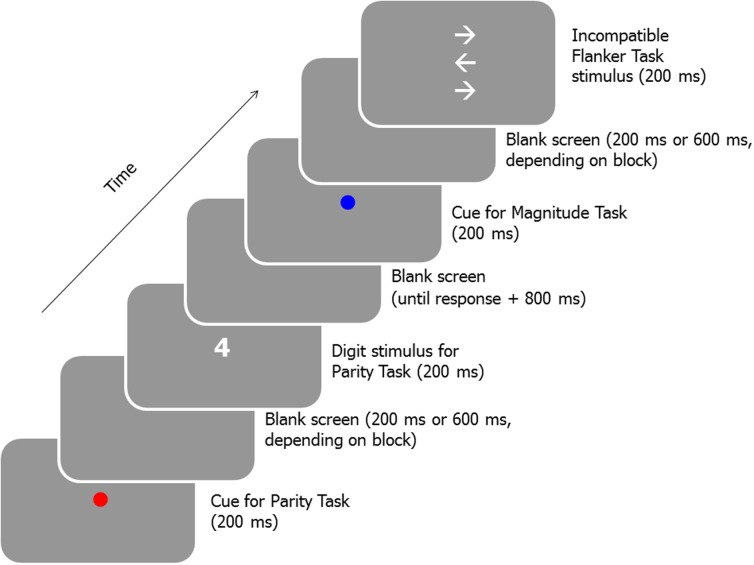
**Schematic of a sequence of a Parity task trial and a Flanker task trial (cued as a magnitude trial) of the experimental blocks of the initial and the final session**.

There were six experimental sessions. One of the participants failed to attend the final session. The interval between two consecutive sessions ranged from 1 to 6 days (mean: 2.63 days). The initial and the final session were structurally identical. In these sessions, participants first received a practice block of 16 flanker task trials. Then, a practice block involving 48 trials of the magnitude and parity task was administered. A third practice block included trials of all three tasks (16 trials of the magnitude and parity task, each, and 8 flanker task trials). A fourth practice block was structurally identical to the subsequent experimental blocks. This block was composed of 96 trials (32 trials of each of the three tasks). On each trial, the task was chosen randomly without replacement and the stimulus was chosen randomly, without replacement, out of the set of possible stimuli of the current task. Flanker task trials were presented with a cue indicating the magnitude task or the parity task with equal probability. Each task cue, digit, and target-flanker ensemble were presented in the center of the screen and displayed for 200 ms. The CTI was set to 800 ms in the practice blocks (with the exception of the first practice block, in which no cues were presented). In the experimental blocks the CTI alternated between 400 and 800 ms from block to block, starting with a 400 ms block. In case of a correct response, the cue of the subsequent trial occurred 800 ms after the response. In case of an incorrect response the message “FALSCHE ANTWORT” (incorrect response) was displayed after a delay of 500 ms in white color for 1000 ms. In case no response was given within 5600 ms (in blocks with a short CTI of 400 ms) or 5200 ms (in blocks with a long CTI of 800 ms) the message “ZU LANGSAM” (too slow) was displayed in white color for 1000 ms. In both cases, the cue of the subsequent trial occurred 800 ms after the offset of the feedback. **Figure 1** displays a schematic of a sequence of trials. Instructions stressed to respond as quickly as possible while attempting to achieve a high level of accuracy. Nine experimental blocks were administered. Between blocks, the participants were allowed to rest for some time.

The training sessions (Sessions 2–5) were identical to the initial and final sessions with the following exceptions. In these sessions the participants were administered only the magnitude task and the parity task. On each trial, each of the two tasks occurred with equal probability and the target digit was chosen randomly from the set of possible digits. Two practice blocks involved 32 trials each (CTI = 800 ms). Then, 10 blocks of 64 trials each were administered. The CTI alternated between 400 and 800 ms from block to block, starting with 800 ms.

## Results

Reaction time and accuracy data of the experimental blocks of the initial and the final session were subjected to statistical analyses. For these analyses, data from the practice blocks, from the first trial of each block, from trials following a flanker task trial, from trials with stimulus repetitions (i.e., the same digit stimulus in the preceding and current trial), and from trials following a trial associated with an incorrect response (i.e., post-errors) were discarded from all analyses. The RT analyses were based only on data from trials with correct responses.

### Digit Tasks

Although our research questions focused on comparisons of performance patterns in the initial session and the final session, we also present the mean RTs and mean error proportions of the digit task trials from the training sessions (i.e., Sessions 2–5). These data are displayed in **Table 1**.

**Table 1:** Mean reaction times (in ms)/mean error percentages (in parentheses: standard deviations) of the digit categorization tasks as a function of session (2–5), task sequence (task repetition vs. task switch), and cue-target interval (CTI: 400 ms vs. 800 ms).

	Task Repetition		Task Switch	
		
	CTI = 400 ms	CTI = 800 ms	CTI = 400 ms	CTI = 800 ms
Session 2	581 (91)/5.1 (4.6)	600 (119)/5.2 (4.1)	643 (124)/7.0 (5.5)	648 (147)/6.9 (6.2)
Session 3	552 (88)/4.5 (4.4)	600 (125)/5.6 (4.1)	613 (131)/7.8 (6.1)	629 (143)/6.8 (6.1)
Session 4	518 (65)/5.3 (3.6)	551 (84)/5.6 (4.9)	574 (94)/7.8 (7.4)	593 (109)/7.9 (6.0)
Session 5	548 (83)/5.3 (4.1)	579 (105)/6.0 (6.1)	597 (96)/8.2 (6.9)	622 (144)/7.8 (7.0)


*CTI, cue-target interval.*

**FIGURE 2:**
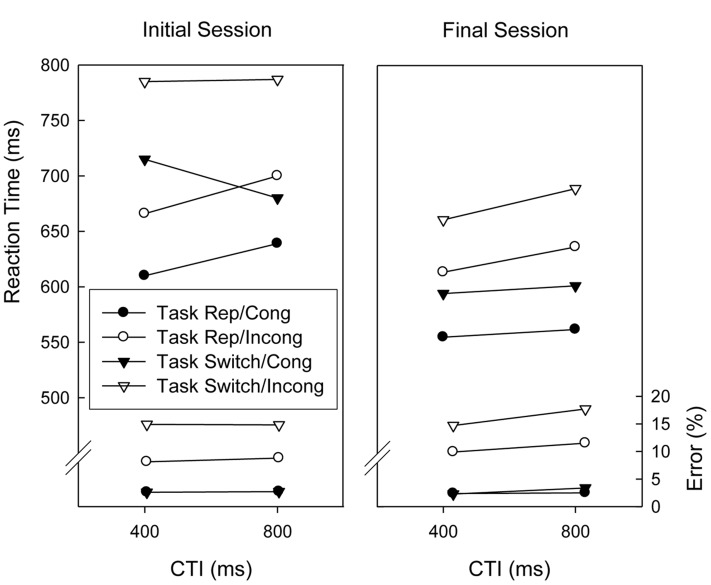
**Mean reaction times and error percentages of the digit categorization tasks before and after extended practice (i.e., initial session and final session) as a function of task sequence, cue-target interval (CTI), and congruency.** Task Rep, Task Repetition; Cong, Congruent stimulus; Incong, Incongruent stimulus.

**Figure 2** displays the results obtained in trials associated with the digit tasks in the initial and final sessions. Analyses of Variance (ANOVAs) with repeated measures on the factors Session (initial vs. final), Task Sequence (repetition vs. switch), CTI (400 ms vs. 800 ms), Congruency (congruent vs. incongruent), and Response Sequence (repetition, switch)^1^ were conducted on the mean RTs and proportions of correct responses. Regarding RTs, there were significant main effects of Session, Task Sequence, and Congruency, *F*(1,18) = 20.7, *p* < 0.01, $\eta_{p}^{2}$ = 0.535, *F*(1,18) = 35.5, *p* < 0.01, $\eta_{p}^{2}$ = 0.664, and *F*(1,18) = 103.2, *p* < 0.01, $\eta_{p}^{2}$ = 0.851, respectively, indicating that responding was slower in the first session than in the final session, slower on task switch trials than on task repetition trials, and slower in incongruent than in congruent trials (i.e., congruency effect). CTI and Congruency interacted, *F*(1,18) = 4.7, *p* < 0.05, $\eta_{p}^{2}$ = 0.20, indicating that the congruency effect tended to be larger with the long CTI (see **Figure 2**). Further, switch costs were smaller in the final session than in the initial session, *F*(1,18) = 7.3, *p* < 0.05, $\eta_{p}^{2}$ = 0.288. This was modulated, however, by a three-way interaction with CTI, *F*(1,18) = 6.5, *p* < 0.05, $\eta_{p}^{2}$ = 0.267, which indicated that the practice-induced reduction of switch costs was confined to the short CTI condition, resulting in the disappearance of the RISC Effect in the final session (see **Figure 2**). Task Sequence also interacted with Response Sequence, *F*(1,18) = 46.1, *p* < 0.01, $\eta_{p}^{2}$ = 0.719, indicating that response repetitions were faster than response switches in task repetition trials but slower in task switch trials. This was further modulated by an interaction with Session, *F*(1,18) = 4.7, *p* < 0.05, $\eta_{p}^{2}$ = 0.206, because the response repetition disadvantage in task switch trials was reduced in the final session.

The analysis of response accuracy yielded significant main effects of Task Sequence and Congruency, *F*(1,18) = 18.7, *p* < 0.01, $\eta_{p}^{2}$ = 0.510, and *F*(1,18) = 60.7, *p* < 0.01, $\eta_{p}^{2}$ = 0.771, respectively, indicating task switch costs and a congruency effect, respectively. Both these factors interacted, *F*(1,18) = 19.6, *p* < 0.01, $\eta_{p}^{2}$ = 0.521, reflecting a larger congruency effect in task switch trials than in task repetition trials. Furthermore, Response Sequence interacted with Task Sequence, *F*(1,18) = 38.4, *p* < 0.01, $\eta_{p}^{2}$ = 0.681, indicating that response repetitions were more error-prone than response switches in task repetition trials versus task switch trials. Response Sequence also interacted with Congruency, *F*(1,18) = 15.0, *p* < 0.01, $\eta_{p}^{2}$ = 0.455, and these three factors (i.e., Response Sequence, Task Sequence, and Congruency) resulted in a significant three-way interaction, *F*(1,18) = 34.9, *p* < 0.01, $\eta_{p}^{2}$ = 0.660. This was because the congruency effect was larger with response repetition trials when the task repeated and larger with response switches when the task switched.

Further, we conducted a control analysis which compared the effects of the task sequence and the CTI for the fifth and the sixth (i.e., final) session, because intermixing trials of the flanker task at the end of practice may have affected processing of the digit tasks in an unknown way, e.g., the probability to switch to a particular digit task was changed from 0.50 to 0.33. (Assuming that practice effects in the digit tasks may have reached an asymptotic level before the final session, a difference in practice between these two sessions can be considered negligible, thus allowing us to attribute any performance difference to the presence vs. absence of flanker task trials.) An ANOVA with repeated measures on the factors Session (fifth vs. sixth/final), Task Sequence (repetition vs. switch), and CTI (400 ms vs. 800 ms), conducted on the mean RTs, yielded only significant main effects of Task Sequence, *F*(1,18) = 27.4, *p* < 0.01, $\eta_{p}^{2}$ = 0.604, and CTI, *F*(1,18) = 9.9, *p* < 0.01, $\eta_{p}^{2}$ = 0.355, indicating switch costs and a general disadvantage when the CTI was long, respectively. The corresponding ANOVA of response accuracy yielded only significant main effects of Task Sequence, *F*(1,18) = 13.0, *p* < 0.01, $\eta_{p}^{2}$ = 0.420, and Session, *F*(1,18) = 6.5, *p* < 0.05, $\eta_{p}^{2}$ = 0.266, indicating switch costs and generally less accurate performance in the final session, respectively. Thus, this data set shows no evidence that the introduction of the Flanker task at the end of practice affects task switching between the digit tasks.

### Flanker Task

**FIGURE 3:**
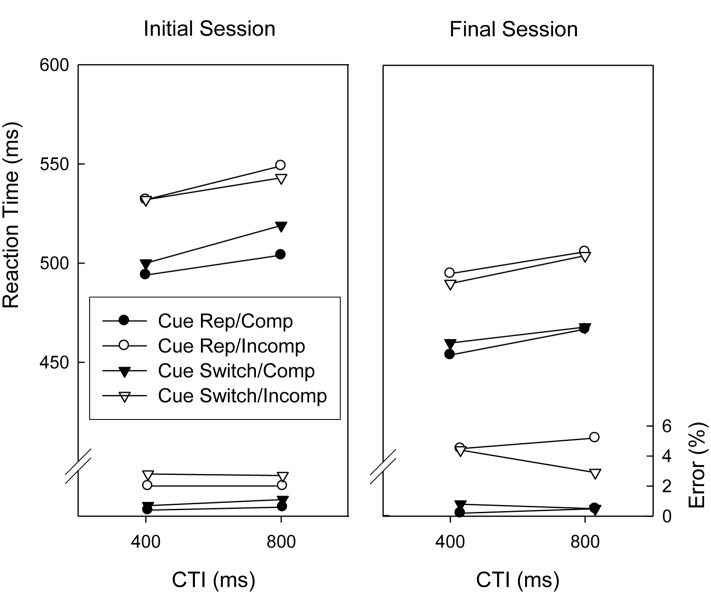
**Mean reaction times and error percentages of the Eriksen flanker task before and after extended practice (i.e., initial session and final session) as a function of cue sequence, flanker compatibility, and cue-target interval (CTI).** Cue Rep, Cue Repetition; Comp, Compatible stimulus; Incomp, Incompatible stimulus.

**Figure 3** displays the results obtained in trials associated with the flanker task. ANOVAs with repeated measures on the factors Session (initial vs. final), Cue Sequence (repetition vs. switch), Flanker Compatibility (compatible vs. incompatible), CTI (400 ms vs. 800 ms), and Response Sequence (repetition, switch) were conducted on the mean RTs and proportions of correct responses of trials involving the flanker task. Note that a cue repetition invalidly indicates a task repetition whereas a cue switch invalidly indicates a task switch. Regarding RTs, there were significant main effects of Session, Flanker Compatibility, and CTI, *F*(1,18) = 30.8, *p* < 0.01, $\eta_{p}^{2}$ = 0.631, *F*(1,18) = 58.1, *p* < 0.01, $\eta_{p}^{2}$ = 0.764, and *F*(1,18) = 17.1, *p* < 0.01, $\eta_{p}^{2}$ = 0.487, respectively, indicating that responding was slower in the initial session than in the final session, slower in incompatible trials than in compatible trials, and slower in the long CTI condition than in the short CTI condition. The only other significant effect was the two-way interaction of Cue Sequence and Flanker Compatibility, *F*(1,18) = 6.0, *p* < 0.05, $\eta_{p}^{2}$ = 0.251, reflecting that the Flanker compatibility effect was larger when the cue indicated a task repetition than when it indicated a task switch.

The corresponding analysis of response accuracy yielded a significant main effect of Flanker Compatibility, *F*(1,18) = 21.2, *p* < 0.01, $\eta_{p}^{2}$ = 0.541, indicating that responses were more error-prone in incompatible trials than in compatible trials. This congruency effect was larger in the final session than in the initial session, indicated by a significant two-way interaction of Flanker Compatibility and Session, *F*(1,18) = 6.4, *p* < 0.05, $\eta_{p}^{2}$ = 0.263. There were also two significant four-way interactions (Session × Cue Sequence × Flanker Compatibility × Response sequence, *F*(1,18) = 4.9, *p* < 0.05, $\eta_{p}^{2}$ = 0.214, and Cue Sequence × Flanker Compatibility × CTI × Response Sequence, *F*(1,18) = 5.8, *p* < 0.05, $\eta_{p}^{2}$ = 0.245) which were not further discussed, however^2^.

## Discussion

The current study aimed at pursuing effects of extended practice on task switching performance, focusing on the optimization of processes of task preparation. In particular, we set out to investigate the occurrence of a previously reported reduction of the RISC Effect after practice (Meiran et al., 2000; see also Cepeda et al., 2001). This investigation extends the assessment of a speed-up of preparation for a task switch under conditions in which task preparation cannot be based on shifting attention toward the perceptual target dimension of the upcoming task, but this switch can be rather based on non-perceptual processes of task preparation.

Performance in the (practiced) digit tasks displayed a monotone trend of RT improvement until the fourth session (see **Table 1**). It thus seems that testing in the sixth session took place under conditions of asymptotic practice benefit. There was also a pronounced improvement for the flanker task despite the fact that this task received no practice during Sessions 2 to 5. Although it can, logically, not be dismissed that the benefit for the flanker task in the final session was brought about by practicing flanker task trials during the initial session (i.e., a test–retest effect; e.g., Green et al., 2014), it is also possible that the higher degree of practice in the other tasks improved the capability of dealing with the occurrence of an unexpected (i.e., invalidly cued) task.

The reduction of the RISC Effect previously reported by Meiran et al. (2000) and—when comparing task switch trials and trials from single-task blocks—Cepeda et al. (2001) was clearly replicated. Given that the tasks with which participants practiced switching in the current study were not associated with perceptually different target dimensions, this finding cannot be attributed to accelerated preparatory shifting of attention toward the stimulus dimension of the upcoming task but must be ascribed to a different component of task-set reconfiguration. It is also worth noting that the reduction of the RISC Effect occurred under conditions of a stimulus set that was twice as large (i.e., eight individual digits) as the stimulus set used by Meiran et al. (2000), demonstrating that the practice-related reduction of the RISC Effect is not confined to very small stimulus sets for which it might be conceivable that increased practice results in a shift from executing different tasks to executing individual stimulus–response translations.

Contrasting with the task switch costs, the congruency effect was not affected by practice, suggesting that task switching practice does not lead to enhanced shielding of task processing from interference exerted by the set of the competitor task. Replicating previous studies (e.g., Rogers and Monsell, 1995; Meiran, 1996) the congruency effect was neither reduced by an increase in preparation time. In fact, in the current study it tended to be larger when the CTI was long.

To gain additional insight into the processing changes brought about by extended task switching practice, we analyzed performance in the flanker task after short and long CTIs (i.e., after preparation for one of the digit tasks). As expected on the assumption of larger malpreparation during a longer CTI, flanker task RTs were generally larger when the CTI was long. The fact that this response slowing after a long CTI was not reduced in the final session seems to cast some doubt on our hypothesis of speeded-up task preparation. If such a speed-up occurred, one might expect that a strong degree of malpreparation would be achieved even after a short CTI, thereby reducing the processing advantage in short CTI trials. It is interesting to note, however, that a general slowing in trials associated with a long CTI also occurred in the digit tasks from Session 2 on (see **Table 1**). Indeed, our additional ANOVA conducted on data of digit task trials from the prefinal and the final sessions (i.e., Sessions 5 and 6, respectively), yielded a significant effect of slower responses when the CTI was long. In light of these findings it seems possible that the expected reduction of response slowing after a long CTI in flanker task trials of the final session was masked by some general (i.e., task-unspecific) slowing.

Flanker compatibility effects were larger when the cue indicated a task repetition (i.e., when the cue matched the previous task and cue) than when the cue indicated a task switch. Various processes have been suggested to account for an increase in flanker interference in different experimental contexts, including, for instance, less selective spatial attention (e.g., Eriksen, 1995), increased spared stimulus processing capacity (and obligatory allocation thereof to the flankers, Lavie, 1995), or increased general response readiness at the time of stimulus presentation (Correa et al., 2010). Although we can only speculate about the precise mechanisms underlying the effect of “executed task-cued task sequence” on the flanker compatibility effect, we would like to point out that it might be linked to recent modeling work of task switching. Specifically, applying diffusion models to task switching performance both Karayanidis et al. (2009) and Schmitz and Voss (2012) found evidence consistent with a lowering of response caution during preparation for a task repetition as compared to a task switch. Given that reduced response caution is liable to increase the relative weight of flanker information (e.g., Gratton et al., 1992), such adjustment might also explain the increase in flanker interference after preparation for a task repetition found in the current study.

Regarding error rates, flanker interference was generally larger in the final session than in the initial session, suggesting the occurrence of more pronounced (susceptibility to) response conflict after task switching practice. Like the unclear role of practice for malpreparation of the probe task, this practice-related increase of susceptibility to irrelevant stimulus objects deserves further investigation.

In general, the present findings are consistent with the literature on practice effects on cognitive control and executive functions, such as working memory updating and dual tasking. In fact, practice demonstrated increased efficiency to update information in working memory. Among others, this increased efficiency is related to an increase in the working memory capacity (e.g., Olesen et al., 2004; Dahlin et al., 2008). In the context of dual tasking, improved executive control functions were related to improved attention allocation between tasks (e.g., Kramer et al., 1995) and attention control skills (e.g., Strobach et al., 2014).

In summary, the current study provides novel evidence for the assumption that task switching practice elicits a speed-up of preparation of non-perceptual processes of task-set reconfiguration. Intermixing trials of a probe task appears to be a useful tool to pinpoint specific components of task processing affected by an experimental intervention, such as practice. Preliminary findings obtained in this study are consistent with the notions of lowered response caution when preparing a task repetition and generally enhanced susceptibility to stimulus-induced conflict after task switching practice.

## Ethics Statement

This study was carried out in accordance with the recommendations of the Declaration of Helsinki of the World Medical Association and approved by the Ethics Committee of the German Psychological Society (Ethik-Kommission der Deutschen Gesellschaft für Psychologie). Informed written consent was obtained from all participants prior to participation.

## Author Contributions

MW and TS planned the experiment and wrote the article. SK programmed the experimental protocol, collected the data, and analyzed the data.

## Conflict of Interest Statement

The authors declare that the research was conducted in the absence of any commercial or financial relationships that could be construed as a potential conflict of interest.

## Footnotes

1 Previous studies, using the same set of responses for both tasks, have yielded a robust interaction of the sequence of responses on consecutive trials with the sequence of tasks. More specifically, whereas task repetitions tend to be facilitated by repetition of the response, the opposite pattern is often found in task switch trials (e.g., Rogers and Monsell, 1995; Meiran, 1996).

2 In an additional analysis, we replaced the factor Response Sequence by the factor Congruency on the Preceding Trial, thus checking for influences of preceding (task) conflict conditions on the flanker compatibility effect. No such influence was found in neither the ANOVA on RTs nor on response accuracy, since none of these analyses revealed two-way interactions of Flanker Compatibility and Congruency on the Preceding Trial as well as no significant higher-order interactions involving these two factors (*F*s < 1). These null findings add to a considerable number of studies suggesting domain-specific mechanisms of attentional adjustment to conflict conditions (overview in Egner, 2008).
